# Controlling Ratios of Plasmid-Based Double Cut Donor and CRISPR/Cas9 Components to Enhance Targeted Integration of Transgenes in Chinese Hamster Ovary Cells

**DOI:** 10.3390/ijms22052407

**Published:** 2021-02-27

**Authors:** Sung Wook Shin, Dongwoo Kim, Jae Seong Lee

**Affiliations:** Department of Molecular Science and Technology, Ajou University, Suwon 16499, Korea; wgend22@ajou.ac.kr (S.W.S.); kdw7258@ajou.ac.kr (D.K.)

**Keywords:** Chinese hamster ovary (CHO), CRISPR/Cas9, double cut donor, homology-directed repair, multiple knock-in, targeted integration, vector construction

## Abstract

Chinese hamster ovary (CHO) cells are the most valuable expression host for the commercial production of biotherapeutics. Recent trends in recombinant CHO cell-line development have focused on the site-specific integration of transgenes encoding recombinant proteins over random integration. However, the low efficiency of homology-directed repair upon transfection of Cas9, single-guide RNA (sgRNA), and the donor template has limited its feasibility. Previously, we demonstrated that a double-cut donor (DCD) system enables highly efficient CRISPR/Cas9-mediated targeted integration (TI) in CHO cells. Here, we describe several CRISPR/Cas9 vector systems based on DCD templates using a promoter trap-based TI monitoring cell line. Among them, a multi-component (MC) system consisting of an sgRNA/DCD vector and Cas9 expression vector showed an approximate 1.5-fold increase in knock-in (KI) efficiency compared to the previous DCD system, when a systematically optimized relative ratio of sgRNA/DCD and Cas9 vector was applied. Our optimization efforts revealed that concurrently increasing sgRNA and DCD components relative to Cas9 correlated positively with KI efficiency at a single KI site. Furthermore, we explored component bottlenecks, such as effects of sgRNA components and applicability of the MC system on simultaneous double KI. Taken together, we improved the DCD vector design by tailoring plasmid constructs and relative component ratios, and this system can be widely used in the TI strategy of transgenes, particularly in CHO cell line development and engineering.

## 1. Introduction

Industrial manufacturing of recombinant therapeutic proteins requires mammalian cells as expression hosts, among which Chinese hamster ovary (CHO) cells are the primary mammalian cells of choice. The cell-line development (CLD) process using CHO cells is based on untargeted random integration of transgenes followed by a selection process and large-scale clone screening [1,2]. While the current CLD platform has generated high producing recombinant CHO cell lines, the process is labor-intensive and causes inconsistent expression of proteins of interest. With the increasing demand for therapeutic proteins in the biopharmaceutical industry with reduced timelines and fewer resources, a more efficient and controllable CLD process is required. Given consistent transgene expression and minimized clonal variation in contrast to that observed in random integration cell lines, site-specific integration of transgenes into desired genomic sites has been implemented in CHO cells as an alternative CLD method [2,3]. 

The emergence of clustered regularly interspaced short palindromic repeats of (CRISPR)/CRISPR-associated protein 9 (Cas9) technology enables precise de novo site-specific integration via homology-directed repair (HDR) pathways in CHO cells upon the generation of double-strand breaks (DSBs) at target genomic sites [3,4]. Homology-mediated targeted integration (TI) results in error-free integration of transgenes into genomic sites of interest, and targeted integrants show reproducible and homogeneous expression of transgenes encoding fluorescent reporter proteins and secreted therapeutic proteins [2,3].

Since the first attempt of TI in CHO cells, several strategies have been developed to improve knock-in (KI) efficiency and shorten the TI-mediated CLD timeline [2]. Owing to the markedly low levels of HDR in CHO cells [2,5], enrichment-based processes, including antibiotic selection and fluorescent enrichment, have been mainly applied to obtain targeted integrants [2,3,6,7,8]. Such enrichment strategies depend on the expression of selection marker genes from donor vector or fluorescence reporter genes from Cas9 and donor vectors.

The CRISPR-Cas9 system is a two-component system consisting of a single guide RNA (sgRNA) and a Cas9 nuclease, and these components are delivered in various formats, either as DNA, RNA or a protein, or directly as a Cas9-gRNA ribonucleoprotein complex [4,9,10]. Together with the CRISPR-Cas9 components, the introduction of DNA repair templates––donor templates with homology sequences of varying length––allows homology-mediated TI. Plasmid DNA-based delivery of CRISPR components and donor templates has been widely used in various applications of CRISPR-Cas9 genome editing because of their ease of use, well-established cloning and transfection methods, and easy availability of plasmid constructs from open-access resources [11]. Compared to embryonic stem cells and models for preclinical and clinical uses, industrial mammalian cells, including CHO cells, are amenable to plasmid transfection and are relatively free of cytotoxicity issues caused by transfection and potential off-target effects from prolonged expression of Cas9 and sgRNA [12]. In addition, plasmid-based delivery offers the ability to add selectable markers, which are necessary for the enrichment of transfected and genome-edited cells.

Traditionally, plasmid-based donor templates have been used to generate large modifications, including the insertion of large transgenes [4,11]. In the context of CLD, the choice of plasmid-based delivery of transgenes in the form of donor templates is the most common for homology-mediated TI [2,3,4,6,7]. In a previous study, we demonstrated that donor templates, termed double-cut donors (DCDs)–which contain the target genomic site sgRNA/protospacer adjacent motif (PAM) recognition sequences outside of homology sequences–markedly increased KI efficiency in CHO cells compared to conventional circular HDR donors [13]. Unlike other in vivo cleavable donor templates with or without micro-homology sequences that can facilitate synchronized target genomic site DSBs and transgene release from its plasmid backbone, DCDs contain rather long homology arm lengths (>150 bp) and display high efficiency and fidelity of complete HDR [14]. Therefore, the use of a DCD enables efficient TI and simultaneous double-transgene KI at two CHO-K1 genomic sites without the selection and enriching processes, reaching a double KI efficiency of 1%–4% [13]. 

Given the prevalence of plasmid vectors for CRISPR-Cas9 mediated site-specific integration and the impact of donor template design, there is still the need, and sufficient room for, further improvement in KI efficiency by modifying the current CRISPR-HDR plasmid vector design. Many design parameters for HDR-mediated TI mainly focus on donor design, including homology arm length and the amount of repair template [15,16,17,18]. Simple titration of the Cas9/gRNA plasmid into the donor template has been shown to increase KI efficiency [15]; however, improvement in vector design by systematically calculating the ratios of the CRISPR-HDR vector components has not been achieved. 

In this study, we described different vector systems for optimized CRISPR/Cas9-mediated TI of transgenes in CHO cells. We observed that elaborate control of the relative ratios of the three key components, Cas9, sgRNA, and the DCD template, is worth considering. Furthermore, we attempted to identify the component bottleneck. Ultimately, with this system, we were able to optimize the previous DCD system and can potentiate enhanced simultaneous TI. 

## 2. Results and Discussion

### 2.1. Effect of Different CRISPR-HDR Vector Configuration on KI Efficiency in CHO Cells

**Figure 1:**
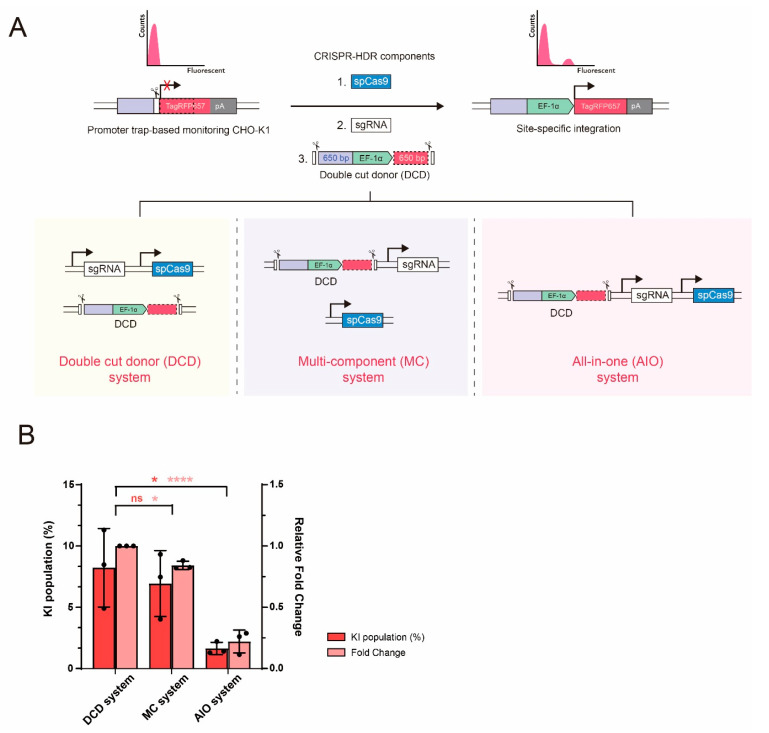
Different vector configurations for CRISPR/Cas9-mediated site-specific integration in CHO cells. (**A**) Schematic illustration of promoter trap-based TI monitoring CHO-K1 cell line and three different systems (DCD, MC*,* and AIO). Introduction of Cas9, sgRNA, and double cut donor (DCD) template into monitoring cell line leads to homology-mediated targeted integration of human EF-1α promoter and restoration of TagRFP657 expression. (**B**) Flow cytometry analysis of DCD, MC*,* and AIO systems 6 days after transfection. The vector constructs were transfected at equal amounts (5:5, *w*/*w*) in the DCD and MC system. Absolute (%) and relative fold changes in TagRFP657^+^ cells were analyzed following the statistical analysis described in the materials and methods section. Relative fold change is normalized to the value obtained with the DCD system. The error bars represent the mean ± standard deviation from three independent experiments. * *p* ≤ 0.05, **** *p* ≤ 0.0001; ns, not significant.

The common plasmid vector configuration for CRISPR-Cas9 mediated site-specific integration is a two-plasmid system consisting of sgRNA/Cas9 in one plasmid and a donor plasmid. Based on the conventional two-plasmid system with an in vivo cleavable DCD template, which is called the DCD system in this study, we constructed two additional plasmid vector systems containing three different CRISPR-HDR components: 1) an all-in-one (AIO) system with expression cassettes for Cas9 and sgRNA and DCD template in one plasmid and 2) a multi-component (MC) system with sgRNA/DCD in one plasmid and a Cas9 expression cassette in another (Figure 1A). To compare KI efficiency among the three vector systems, we used a promoter trap-based TI monitoring CHO-K1 cell line [13], in which the TI of the promoter from the donor templates restored the expression of TagRFP657, followed by quantitative measurement using flow cytometry (Figure 1A). The introduction of three vector systems by electroporation led to the detection of TagRFP657^+^ KI populations. Interestingly, the AIO system showed the lowest KI efficiency, while the DCD and MC systems exhibited comparable KI efficiencies (Figure 1B). Compared to the DCD and MC systems, which use two plasmids, a single plasmid-based AIO system can offer advantages for multiple and simultaneous targeting [19]. However, the AIO plasmid may be self-cleaved by the Cas9/sgRNA complex because of the two sgRNA/PAM recognition sites present in the plasmid as a part of the DCD template, which adversely affects the expression and integrity of the components [20].

### 2.2. CRISPR-HDR Component Titration and Ratio Optimization in the DCD and MC Systems

**Figure 2:**
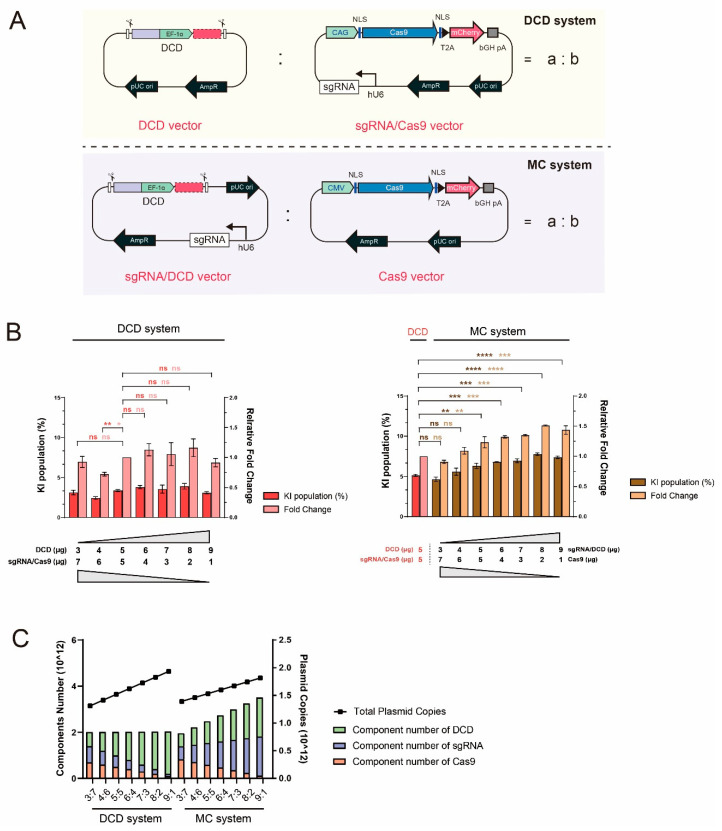
Enhancement of knock-in (KI) efficiency via multi-component (MC) system with optimized CRISPR component ratio. (**A**) Detailed schematic diagram of the vector construction of the DCD and MC systems. The main difference between the two systems is the location of the sgRNA expression cassette: either in the Cas9 vector (sgRNA/Cas9) or the DCD template (sgRNA/DCD) in the DCD and MC system, respectively. In the case of the sgRNA/DCD vector, the ampicillin resistance gene and origin of replication separate the DCD template and sgRNA expression cassette at a distance. (**B**) Evaluation of the KI efficiency of different ratios of CRISPR-HDR components in DCD (left) and MC systems (right). Seven different ratios (*w*/*w*) from 3:7 to 9:1 were tested in both systems, as indicated in a:b (A). The previous DCD 5:5 (*w*/*w*) system was set as a relative control in both systems. The error bars represent the mean ± standard deviation from two or three independent experiments. *n* = 3 for the DCD system and *n* = 2 for the MC system, * *p* ≤ 0.05, ** *p* ≤ 0.01, *** *p* ≤ 0.001, **** *p* ≤ 0.0001; ns, not significant. (**C**) Calculation of plasmid copies and the number of CRISPR–HDR components tested in (B).

Following the initial evaluation of plasmid vector systems, we used the DCD and MC systems for the remainder of this study. As observed in the AIO system (Figure 1B), self-cleavage sites negatively affected component expression. However, one of the two plasmids constituting the MC system contained both a DCD template and an sgRNA expression cassette, which were juxtaposed with a minimum-linker sequence (10 bp) (Figure 1A). As the short-spacer sequence may not be optimal for the buffering of sgRNA expression cassettes against degradation by nucleases upon self-cleavage of the plasmid, we reconstructed the backbone of the plasmid and placed the DCD template between the origin of replication and ampicillin resistance expression cassette (Figure 2A). 

Next, to determine whether changes in the ratio of CRISPR-Cas9 to donor components can affect KI efficiency, the TI monitoring CHO-K1 cell lines were transfected with seven different ratios (*w*/*w*) of either sgRNA/Cas9 plasmid to the DCD plasmid for the DCD system or sgRNA/DCD plasmid to the Cas9 plasmid for the MC system (Figure 2B). We found that increasing the level of sgRNA/DCD plasmid led to a gradual increase in KI efficiency in the MC system, except for a 9:1 ratio of sgRNA/DCD plasmid to the Cas9 plasmid. The highest KI efficiency was obtained at an 8:2 (*w*/*w*) ratio, corresponding to a 1.5-fold increase compared with the DCD 5:5 (*w*/*w*) system (*p* ≤ 0.0001). In contrast to the MC system, no significant change in KI efficiency was observed in the DCD system, according to the change in plasmid ratio (Figure 2B). Calculation of the number of components for different plasmid ratios in each system revealed that the number of DCD templates and Cas9 expression cassettes was similar, but that of the sgRNA expression cassette was distinctly different in both systems at each plasmid ratio (Figure 2C and Table S1). In both systems, changing the ratio (*w*/*w*) from 3:7 to 9:1 increased the number of DCD templates while decreasing the amount of Cas9 expression cassettes. Given the positive results observed only in the MC system, these results suggest that increasing the levels of both the sgRNA and DCD templates with minimal use of Cas9 will improve KI efficiency. The same trend was also observed targeting another locus which supports the result regardless of the integration site (Figure S1 and Table S1). The minimum requirement of Cas9 expression cassettes can be supported by recent studies on Cas9 cytotoxicity and cell cycle arrest [21,22,23]. Genome editing by Cas9 can induce p53-mediated G1 cell cycle arrest, which in turn decreases the HDR rate, as well as the toxic response in human cells [21,22,23]. In addition, persistent Cas9 binding to DNA after cleavage may hinder DSB sites from being recognized by repair machinery [23]. Collectively, these data indicate that the balanced use of CRISPR-HDR components through the titration of the MC system led to an increase in KI efficiency. 

### 2.3. Limited Effect of sgRNA Supplementation in the MC System

**Figure 3:**
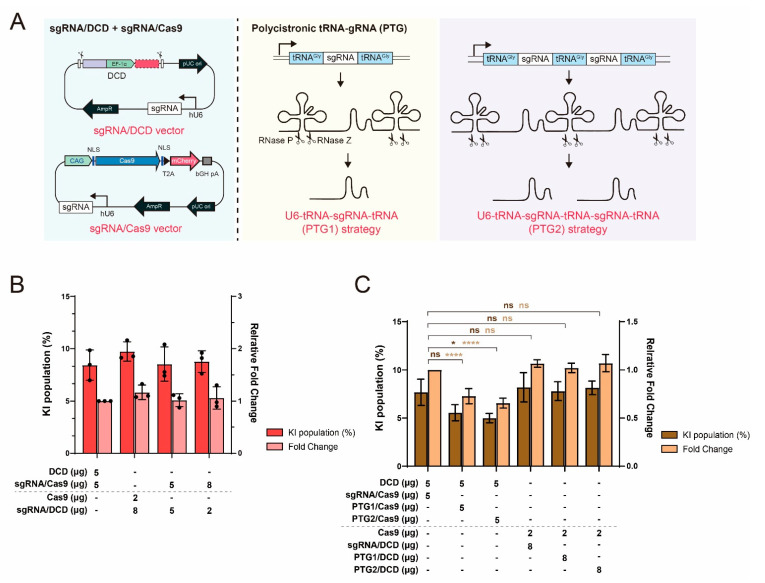
Limited effect of sgRNA supplementation on knock-in (KI) efficiency. (**A**) Schematic diagram of vector constructs needed for sgRNA supplementation. To increase the component number of sgRNA, the sgRNA/DCD plasmid in the multi-component (MC) system and sgRNA/Cas9 plasmid in the DCD system were introduced together, or synthetic polycistronic–tRNA–gRNA (PTG) genes consisting of tandemly arrayed tRNA–gRNA units were inserted into the sgRNA expression cassettes downstream of the U6 promoter. (**B**) The combined use of the MC and DCD systems. The sgRNA/DCD and sgRNA/Cas9 vectors used in the MC and DCD systems, respectively, were transfected at a ratio of 5:5 (*w*/*w*) or 8:2 (*w*/*w*). The previous DCD (5:5, *w*/*w*) and optimized MC (8:2, *w*/*w*) were also tested to assess the effect of their combined use on KI efficiency. By the statistical analysis, these results are indicated as non-significant. (**C**) Application of the PTG system to the DCD and MC systems. The error bars represent the mean ± standard deviation from three independent experiments. * *p* ≤ 0.05, **** *p* ≤ 0.0001; ns, not significant.

As increasing the amount of sgRNA and DCD template was effective in the MC system, further increasing the amount of both components may be beneficial to improve KI efficiency. In the case of the DCD template, it was limited in order to further increase the amount, except by increasing the amount of total plasmid during transfection or reducing the size of the donor. However, the amount of sgRNA can be increased while maintaining the current format (Figure 3A). The combined use of the sgRNA/DCD plasmid in the MC system and sgRNA/Cas9 plasmid in the DCD system can provide additional sgRNAs compared with the previous MC system. According to the calculation, an approximate 13% increase in the amount of sgRNA could be expected in the 8:2 (*w*/*w*) ratio of sgRNA/DCD plasmid to sgRNA/Cas9 plasmid (Figure S2 and Table S1). We also harnessed a polycistronic–tRNA–gRNA (PTG) strategy, which was developed to produce numerous gRNAs from synthetic genes with tandemly arrayed tRNA–gRNA architecture via the endogenous tRNA-processing system [24,25]. This system allows simultaneous expression of multiple gRNAs and boosts Pol III transcription, leading to higher expression of gRNA [24,25]. Compared to other multiple gRNA expression systems, the PTG system relies on the endogenous tRNA-processing system without additional exogenous components, and the size of additional tRNA sequences is small, rather than introducing multiple gRNA expression cassettes [25]. Two PTG formats with a structure of tRNA–sgRNA–tRNA (PTG1) and tRNA–sgRNA–tRNA–sgRNA–tRNA (PTG2) were designed and inserted into the sgRNA/Cas9 plasmid in the DCD system and sgRNA/DCD plasmid in the MC system. 

Although two sgRNA supplementation approaches were likely to provide additional sgRNAs based on our calculations, we did not observe significant improvements in KI efficiency, suggesting that increasing sgRNA alone may not be sufficient to increase the targeting efficiency (Figure 3B and Figure S2). Moreover, the application of the PTG strategy lowered KI efficiency regardless of the integration site, even with the replacement of sgRNA with PTG1 (Figure 3C and Figure S3). When using multiple sgRNA expression systems, additional 5′ and 3′ end processing is required to produce mature multiple gRNAs. In the present PTG system, we applied glycine tRNA of rice. A previous study demonstrates that the tRNA processing efficiency of exogenous tRNA sequences, such as rice tRNA^Gly^, is not efficient in human cells compared to the endogenous one although tRNA processing is conserved across kingdoms [26]. Therefore, the use of the CHO endogenous tRNA sequence for the PTG strategy could improve processing efficiency, leading to efficient gRNA production. Alternatively, a different multiplex gRNA expression strategy, such as the Drosha-mediated gRNA-shRNA structure [27], can be utilized. The gRNA and shRNA transcript is cleaved by endogenous ribonuclease III (Drosha), into functional gRNAs and shRNAs. By doing so, multiple gRNAs and shRNAs that silence the expression of proteins related to non-homologous end joining can be generated to further increase HDR efficiency.

Together with increased sgRNA production, a concurrent increase in the DCD template could resolve the limited effect shown in Figure 3. Because the length of homology arms can be reduced in the DCD system without adverse effects on HDR efficiency and KI patterns [14], the amount of DCD template could be increased by approximately 17% by reducing the current 650 bp homology arms to 300 bp. In addition to increasing the absolute component amount, improved delivery of sgRNA/DCD plasmid into the nucleus would have a synergistic effect. By introducing DNA nuclear targeting sequences such as the SV40 enhancer region [28] and tandem NFκB binding sites [29] in the sgRNA/DCD plasmid, nuclear import of the plasmid DNA can be facilitated, which will aid in the increased availability of donor and sgRNA ready for Cas9/sgRNA complex formation in the nucleus. 

### 2.4. Application of Simultaneous MC–TI and Split-Intein MC System on Double TI 

**Figure 4:**
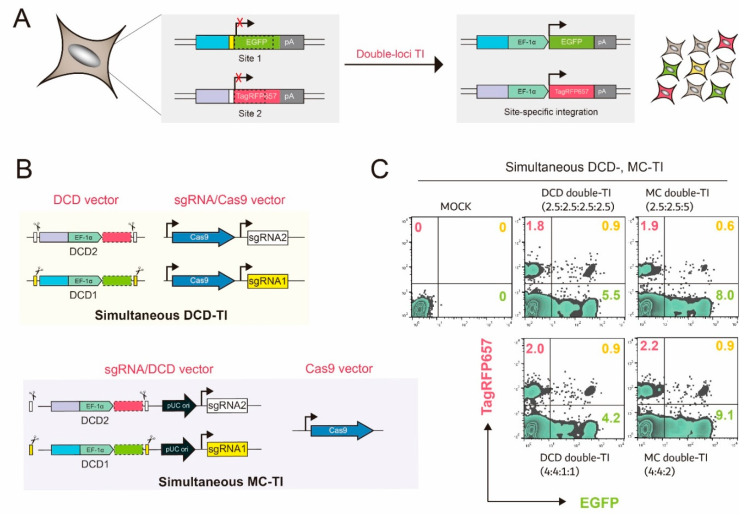
Application of the multi-component (MC) system to double-targeted integration (TI). (**A**) Schematic diagram of promoter trap-based double-TI monitoring CHO-K1 cell line and (**B**) vector constructs needed for double TI in DCD and MC system. The two genomic site-specific sgRNA/protospacer adjacent motif sequences were included in the corresponding targeting DCD templates (DCD1 and DCD2). (**C**) Simultaneous DCD- and MC-TI. Following 6-day post-transfection of all vector constructs at once, EGFP and TagRFP657 expression levels were quantified using flow cytometry. Two different ratios (*w*/*w*) were tested in each DCD and MC system and the simultaneous DCD–TI (2.5:2.5:2.5:2.5, *w*/*w*/*w*/*w*) was set as a control value. Representative dot plot images from three independent experiments are shown with mean values. *n* = 3.

Next, to extend our findings, we applied an optimized MC 8:2 (*w*/*w*) system to integrate exogenous genes of interest into double loci using the double TI monitoring CHO-K1 cell line [13]. Briefly, apart from the TagRFP657-based reporter cassette (site 2), the EGFP-based one (site 1) was also integrated into another genomic site of CHO-K1 to detect single and double TI events (Figure 4A and Table S2).

To construct CRISPR-HDR components, DCD and MC systems require different numbers of vector constructs (Figure 4B). Accordingly, to compare the applicability of the two systems to double TI, we set the previous simultaneous DCD–TI [13] at a ratio of 2.5:2.5:2.5:2.5 (*w*/*w*/*w*/*w*) for DCD2, DCD1, sgRNA2/Cas9, and sgRNA1/Cas9 as a reference value (Figure 4C). Because TagRFP657 (site 2)––as well as EGFP (site1)-based single-site MC titration––showed maximal KI efficiency at a ratio of 8:2 for sgRNA/DCD and Cas9 vector (Figure 2B and Figure S1A), a ratio of 4:4:2 (*w*/*w*/*w*) for sgRNA2/DCD2, sgRNA1/DCD1, and Cas9 vector was tested by providing two sgRNA/DCDs in equal amounts while maintaining minimal use of the Cas9 vector (Figure 4C). Furthermore, simultaneous DCD–TI (4:4:1:1, *w*/*w*/*w*/*w*) and simultaneous MC–TI (2.5:2.5:5, *w*/*w*/*w*), corresponding to simultaneous MC–TI (4:4:2, *w*/*w*/*w*) and simultaneous DCD-TI (2.5:2.5:2.5:2.5, *w*/*w*/*w*/*w*), respectively, was also conducted (Figure 4C). All simultaneous DCD– and MC–TIs exhibited a similar percentage to EGFP/TagRFP657 double-positive populations by approximately 0.9%, except for simultaneous MC–TI (2.5:2.5:5, *w*/*w*/*w*), which was approximately 0.6% (*p* ≤ 0.01). This result is consistent with our data showing that the number of sgRNA and DCD changes depending on the number of sgRNA/DCD vectors (Figure 2C, Figure S4, and Table S1), and these changes are directly related to KI efficiency within the MC system between MC–TI (4:4:2, *w*/*w*/*w*) and MC–TI (2.5:2.5:5, *w*/*w*/*w*). Interestingly, although the simultaneous MC–TI (4:4:2, *w*/*w*/*w*) showed a comparable level of double TI integrants with simultaneous DCD–TI (2.5:2.5:2.5:2.5, *w*/*w*/*w*/*w*), 1.2- to 1.6-fold increase in TagRFP657 or EGFP single positive populations was detected, which implies that the potential for these populations to move onto double KI through further studies. For example, because the absolute KI efficiency of the TagRFP657 reporter (site 2) was generally lower than that of the EGFP reporter (site 1)––possibly due to the sgRNA editing efficiency and genomic environment of the integration site [30,31]––systematic adjustment efforts between multiple sgRNA/donor vector ratios may lead to non-biased single-KI efficiency and, eventually, to improved double-TI intersection.

## 3. Materials and Methods

### 3.1. Plasmid Design and Construction

The plasmids created and used in this study are listed in Table S3. Based on the sgRNA/Cas9 plasmids and donor plasmids in the DCD system [13], all the plasmids, except for sgRNA plasmids containing PTG structures, were constructed via the uracil-specific excision reagent (USER) cloning method [3]. DNA bricks were generated by PCR amplification using Phusion U Hot Start DNA polymerase (Thermo Fisher Scientific, Waltham, MA, USA), followed by purification from 1% agarose TAE gel with the NucleoSpin^®^ Gel and PCR Cleanup Kit (Macherey-Nagel, Duren, Germany). The resulting DNA bricks were assembled with USER enzyme (New England Biolabs, Ipswich, MA, USA), and the assembled DNA bricks were transformed into *Escherichia coli* Mach1 competent cells (Thermo Fisher Scientific). The list of DNA bricks and PCR templates used for each construct is summarized in Table S4. 

To construct the PTG containing vectors, the Bve1 recognition sequence–tRNA–Bbs1 recognition sequence–sgRNA scaffold–tRNA–Bve1 recognition sequence was generated through PCR amplification using Phusion High-Fidelity PCR Master Mix (Thermo Fisher Scientific) and digested with Bve1 (Thermo Fisher Scientific). The digested insert was cloned into pU6–(BbsI)_CBh–Cas9–T2A–mCherry (Addgene plasmid #64324) using T4 DNA ligase (Thermo Fisher Scientific), resulting in the PTG/Cas9–2A–mCherry plasmid. Then, sgRNA expression cassettes containing PTG structures were amplified from the PTG/Cas9–2A–mCherry plasmid, which were used to construct the PTG/donor plasmid in the MC system via USER cloning. Based on the PTG/Cas9–2A–mCherry plasmid and PTG/donor plasmid, duplexes of single-stranded oligos of sgRNA1 and sgRNA2 were inserted to generate PTG1 containing plasmids. In the case of the PTG2-containing vectors, Bbs1 recognition sequence–sgRNA1 or 2-tRNA–sgRNA1 or 2-Bbs1 recognition sequence was generated through PCR amplification. After digestion with Bbs1 (New England Biolabs), the insert was ligated into the PTG/Cas9–2A-mCherry plasmid and PTG/donor plasmid, resulting in PTG2 containing plasmids. The transformation method is mentioned above. 

For transfection, sequence-verified plasmids were purified using the NucleoBond Xtra Midi EF kit (Macherey-Nagel) according to the manufacturer’s instructions.

### 3.2. Cell Lines and Cell Culture 

The CHO-K1 cell line containing promoter-less reporter fluorescent protein expression cassettes, a TI monitoring CHO-K1 cell line, was generated in a previous study [13]. The cell line was maintained in Dulbecco’s modified Eagle’s medium (Gibco, Gaithersburg, MD, USA) supplemented with 10% fetal bovine serum (Hyclone, Logan, UT, USA), 500 μg/mL G418 (Sigma Aldrich, St. Louis, MO, USA), and 3 μg/mL puromycin (Sigma Aldrich), and incubated at 37 °C in a humidified 5% CO_2_ atmosphere. Viable cell density and viability were measured using a Countess II FL automated cell counter (Invitrogen, Carlsbad, CA, USA) and the trypan blue-dye exclusion method. 

### 3.3. KI Efficiency Measurement Using Flow Cytometry 

The promoter trap-based TI monitoring CHO-K1 cell line was transfected with various plasmid combinations at the ratios indicated in the text. The transfection was performed using a NEPA21 electroporator (Nepagene, Chiba, Japan) as per the transfection procedures described previously [13]. At 72 h post-transfection, the cells were subcultured at a concentration of 2 × 10^5^ cells/mL for an additional 3 days. For flow cytometry analysis, the cells were harvested and resuspended in phosphate-buffered saline supplemented by 10% fetal bovine serum (Hyclone). The percentage of TagRFP657^+^ or EGFP^+^ populations was measured using FACSCalibur (Becton Dickinson, NJ, USA), and the results were analyzed using FlowJo software (Tree Star, OR, USA).

### 3.4. Plasmid Copy Number Calculation 

Depending on the length and weight of the plasmid, its copy number was calculated as shown.

(1) $$\mathrm{Plasmid}\;\mathrm{copy}\;\mathrm{number}\;=\frac{\left(\mathrm{weight}\;\left(\mathrm{ng}\right)\;\times \;6.022\times 10^{23}\right)}{\left(\mathrm{length}\;\left(\mathrm{bp}\right)\;\times \;10^{9}\;\times \;650\right)}$$

where one mole of a nucleotide base pair weighs 650 g, and Avogadro’s number is molecules/mole. The molecular weight of the double-stranded DNA template was estimated by multiplying its length (bp) by 650. The number of each component was calculated by multiplying the plasmid copy number by the component number in each plasmid.

### 3.5. Statistical Analysis

Statistical analysis was performed using one-way analysis of variance, followed by Dunnett’s multiple comparisons test. *p*-values were calculated using GraphPad Prism software (GraphPad Software, San Diego, CA, USA), and *p* ≤ 0.05 was considered significant. (* *p* ≤ 0.05, ** *p* ≤ 0.01, *** *p* ≤ 0.001, and **** *p* ≤ 0.0001).

## 4. Conclusions

Based on the previous DCD system, we modified the CRISPR–HDR plasmid vector design to further optimize the relative ratio of CRISPR–HDR components. The MC system with an optimized 8:2 ratio (*w*/*w*) of sgRNA/DCD plasmid to the Cas9 plasmid-enhanced KI efficiency at single integration sites compared with the DCD system. The MC system has several advantages for homology-directed TI using CRISPR/Cas9 over the DCD system: (1) simultaneous increase in sgRNA and donor template leading to improved KI efficiency; (2) minimal use of Cas9 enabling less-toxic effects on cells; and (3) reduction of the number of plasmid constructs per integration site required for multiple gene KI. By setting our optimized MC vector system as a basal configuration, there is room to further increase the probability of acquiring targeted integrants with well-published strategies, such as chemical treatment, effector gene expression or fusion Cas9, and cell cycle synchronization [2]. The present MC system had similar double KI efficiency to the DCD system; however, the improved KI efficiency at a single site exhibited the potential of the MC system. In conclusion, we demonstrated the significance of the relative ratios of CRISPR-HDR components in plasmids for KI efficiency. The present optimized plasmid-vector constructs provide an easy-to-apply plasmid design and could streamline mammalian cell line development and cell engineering.

## Data Availability

Data is contained within the article or supplementary material.

## Supplementary Materials

Supplementary Materials can be found at https://www.mdpi.com/1422-0067/22/5/2407/s1.
