# Supplementary material for: Controlling Ratios of Plasmid-Based Double Cut Donor and CRISPR/Cas9 Components to Enhance Targeted Integration of Transgenes in Chinese Hamster Ovary Cells
Source: Int J Mol Sci. 2021 Feb 27;22(5):2407. doi: 10.3390/ijms22052407 (PMC7957797; doi:10.3390/ijms22052407)
Supplement: Supplementary file 1 [file ijms-22-02407-s001.pdf]

Supplementary Material

**Section, figures and tables**	**Page number**
Supplementary Figure S1	2
Supplementary Figure S2	3
Supplementary Figure S3	4
Supplementary Figure S4	5
Supplementary Table S1	6
Supplementary Table S2	8
Supplementary Table S3	9
Supplementary Table S4	12
Supplementary References	14

FIGURES

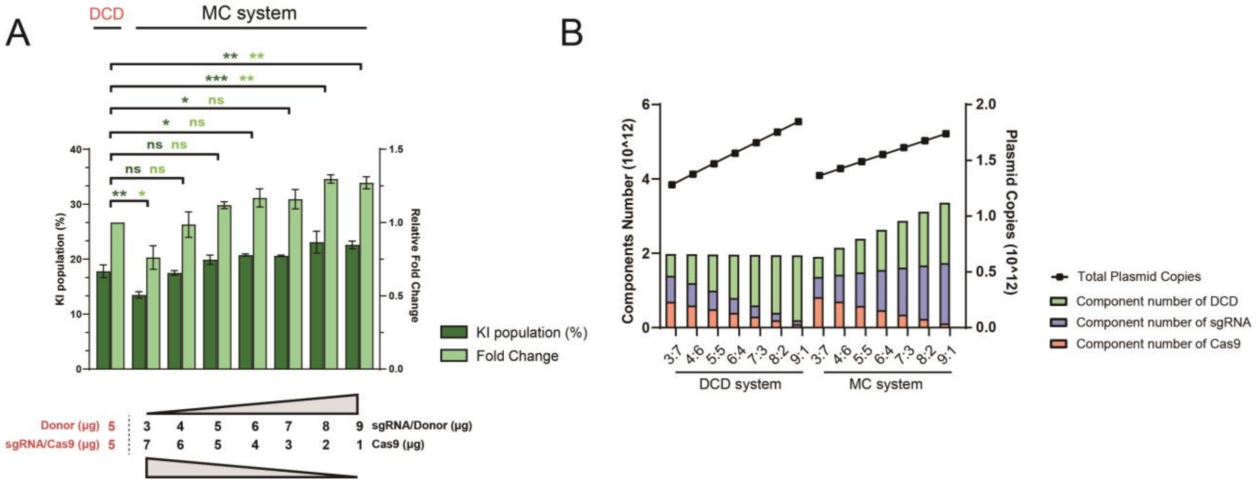

Supplementary Figure S1. Titration of multi-component (MC) system with different ratios of constructs. (A) Evaluation of the knock-in (KI) efficiency of *MC* system in another locus (site 1, EGFP reporter) using double TI monitoring CHO-K1 cell line. The seven differnt ratios of sgRNA/DCD plasmid to Cas9 plasmid (from 3:7 to 9:1, w/w) were tested in *MC* system and the previous *DCD* 5:5 (w/w) system was set as a referecne value. The error bars represent the mean ± standard deviation from two independent experiments, $*p \leq 0.05$, $**p \leq 0.01$, $***p \leq 0.001$; ns, not significant. (B) Calculation of plasmid copies and the number of CRISPR-HDR components tested in (A).

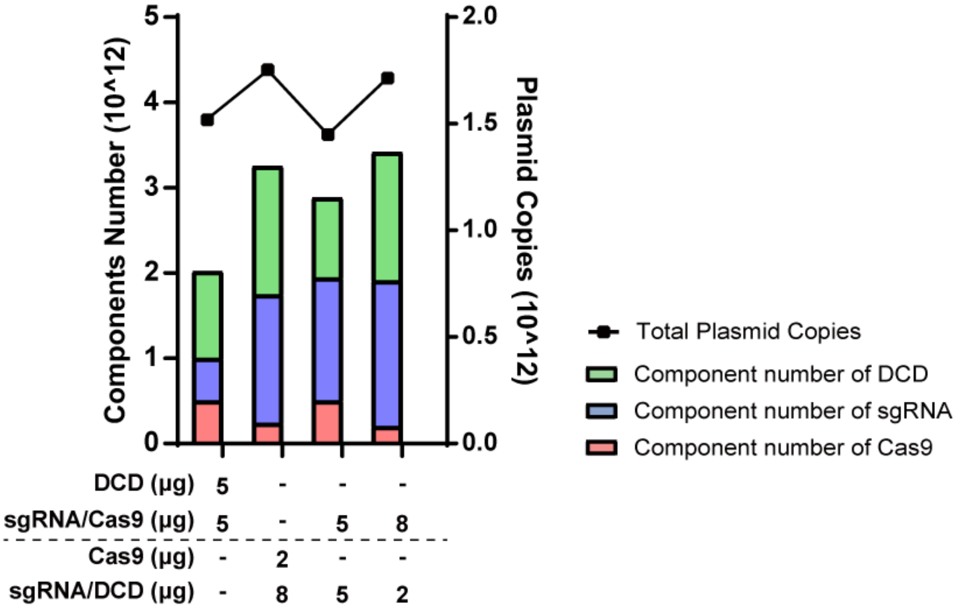

Supplementary Figure S2. Calculation of the number of CRISPR-HDR components and plasmid copies used in application of combined *MC* and *DCD* systems. For TI test using the combination of *MC* and *DCD* systems, sgRNA/DCD vector in the *MC* system and sgRNA/Cas9 vector in the *DCD* system were transfected at a ratio of 5:5 and 8:2, respectively. The previous *DCD* (5:5, w/w) and optimized *MC* (8:2, w/w) were also tested as controls to evaluate the effect of the combined use on KI efficiency.

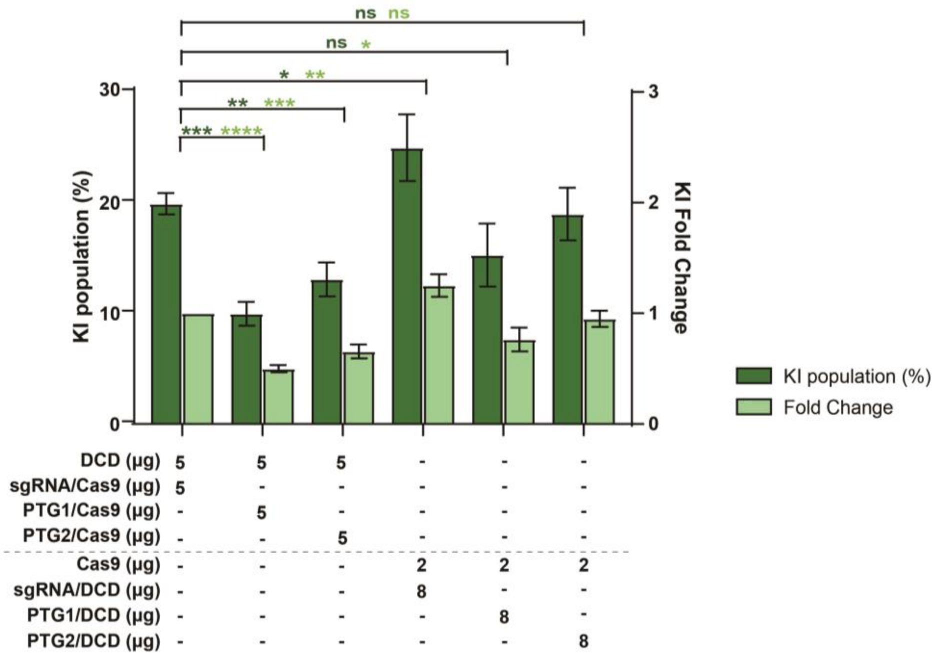

Supplementary Figure S3. Supplementation of the single guide RNA expression using polycistronic-tRNA-gRNA (PTG) strategy. The PTG system was applied to another locus (site 1, EGFP reporter) using double TI monitoring CHO-K1 cell line. Two PTG formats of tRNA-gRNA-tRNA (PTG1) and tRNA-gRNA-tRNA-gRNA-tRNA (PTG2) were tested with double cut donor (DCD) and *MC* system. The error bars represent the mean ± standard deviation from three independent experiments; $*p \leq 0.05$, $**p \leq 0.01$, $***p \leq 0.001$, $****p \leq 0.0001$; ns, not significant.

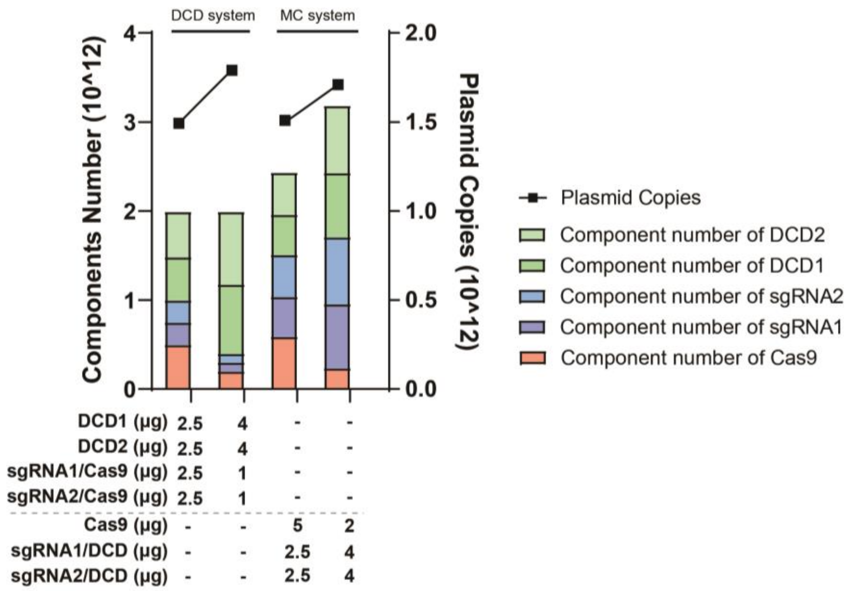

Supplementary Figure S4. Calculation of the number of CRISPR-HDR components and plasmid copies in the double targeted integration (TI) experiment. For simultanoues TI using *DCD* and *MC* system, the ratio of 2.5:2.5:2.5:2.5 and 4:4:1:1 (w/w/w/w) for DCD1, DCD2, sgRNA1/Cas9, and sgRNA2/Cas9 vector in *DCD* system and 2.5:2.5:5 and 4:4:2 (w/w/w) for sgRNA1/DCD1, sgRNA2/DCD2, and Cas9 vector in *MC* system were tested using double TI monitoring cell line. The components number and plasmid copies were calcuated in each conditions.

TABLES

Supplementary Table S1. Calculation of component number in the *DCD* and *MC* system

Vector system	Ratio (w/w) of transfected plasmid	sgRNA1 expression cassette (10^{12})	Cas9 expression cassette (10^{12})	Double cut donor 1 (10^{12})
***DCD* system** (DCD1 : sgRNA1/Cas9-2A-mCherry)	3 : 7	0.698	0.698	0.582
	4 : 6	0.599	0.599	0.776
	5 : 5	0.499	0.499	0.970
	6 : 4	0.399	0.399	1.164
	7 : 3	0.299	0.299	1.358
	8 : 2	0.200	0.200	1.553
	9 : 1	0.100	0.100	1.747
***MC* system** (sgRNA1/DCD1 : MC_Cas9-2A-mCherry)	3 : 7	0.540	0.824	0.540
	4 : 6	0.720	0.706	0.720
	5 : 5	0.901	0.588	0.901
	6 : 4	1.081	0.471	1.081
	7 : 3	1.261	0.353	1.261
	8 : 2	1.441	0.235	1.441
	9 : 1	1.621	0.118	1.621

Vector system	Ratio (w/w) of transfected plasmid	sgRNA2 expression cassette (10^{12})	Cas9 expression cassette (10^{12})	Double cut donor 2 (10^{12})
***DCD* system** (DCD2 : sgRNA2/Cas9-2A-mCherry)	3 : 7	0.698	0.698	0.611
	4 : 6	0.599	0.599	0.815
	5 : 5	0.499	0.499	1.019
	6 : 4	0.399	0.399	1.222
	7 : 3	0.299	0.299	1.426
	8 : 2	0.200	0.200	1.630
	9 : 1	0.100	0.100	1.833

***MC* system** (sgRNA2/DCD2 : MC_Cas9-2A-mCherry)	3 : 7	0.565	0.824	0.565
	4 : 6	0.754	0.706	0.754
	5 : 5	0.942	0.588	0.942
	6 : 4	1.131	0.471	1.131
	7 : 3	1.319	0.353	1.319
	8 : 2	1.508	0.235	1.508
	9 : 1	1.696	0.118	1.696
sgRNA2/DCD2 : sgRNA2/Cas9-2A-mCherry	5 : 5	1.441	0.499	0.942
	8 : 2	1.707	0.200	1.508

**Vector system**	**Ratio (w/w/w/w) of transfected plasmid**	**sgRNA2 (10^{12})**	**sgRNA1 (10^{12})**	**Cas9 expression cassette (10^{12})**	**Double cut donor 2 (10^{12})**	**Double cut donor 1 (10^{12})**
***DCD* system** (DCD1 : DCD2 : sgRNA1/Cas9-2A-mCherry : sgRNA2/Cas9-2A-mCherry)	2.5 : 2.5 : 2.5 : 2.5	0.249	0.249	0.485	0.499	0.509
	4 : 4 : 1 : 1	0.100	0.100	0.776	0.200	0.815
***MC* system** (sgRNA1/DCD1 : sgRNA2/DCD2 : MC_Cas9-2A-mCherry)	2.5 : 2.5 : 5	0.471	0.450	0.450	0.588	0.471
	4 : 4 : 2	0.754	0.720	0.720	0.235	0.754

Supplementary Table S2. Integration site information

Internal name (Integrated reporter)	Scaffold [1,2]	Genomic location	Upstream gene	Downstream gene	Chromatin state [1,2]
Site 1 (EGFP)	KE682437	Intergenic	Erc1	Rad52	Weak enhancer (state number 6)<br>- enriched mark: H3K4me1, H3K27ac low<br>Strong enhancer (state number 7)<br>– enriched mark: H3K27ac high<br>Active promoter (state number 9)<br>– enriched mark: H3K4me3, H3K27ac high<br>Flanking active TSS (state number 11)<br>– enriched mark: H3K4me3, H3K4me1
Site 2 (TagRFP657)	KE685893	C1galt1c1	LOC113837817	Mcts1	Strong transcription (state number 4)<br>– enriched mark: H3K36me3<br>Weak enhancer (state number 6)<br>- enriched mark: H3K4me1, H3K27ac low<br>Strong enhancer (state number 7)<br>– enriched mark: H3K27ac high<br>Active promoter (state number 9)<br>– enriched mark: H3K4me3, H3K27ac high<br>Active promoter (state number 10)<br>– enriched mark: H3K4me3, H3K27ac low<br>Flanking active TSS (state number 11)<br>– enriched mark: H3K4me3, H3K4me1

Supplementary Table S3. Plasmids used in this study

System	Plasmid name	Description	Size (bp)	Reference
-	Cas9-2A-mCherry	Cas9 2A peptide-linked mCherry without specific targeting sgRNA expression control (Addgene #64324)	9,283	[3]
DCD system	sgRNA1/Cas9-2A-mCherry	Cas9 2A peptide-linked mCherry and sgRNA1 expression vector targeting monitoring cell line (EGFP upstream)	9,286	[4]
	sgRNA2/Cas9-2A-mCherry	Cas9 2A peptide-linked mCherry and mouse Rosa26_sgRNA expression vector targeting double KI monitoring cell line (TagRFP657 upstream)	9,286	[4]
	DCD1	Human EF-1α double cut donor targeting monitoring cell line (Site 1)	4,774	[4]
	DCD2	Human EF-1α double cut donor targeting monitoring cell line (Site 2)	4,548	[4]
MC system (Original backbone)	MC_Cas9-2A-mCherry	Cas9 2A peptide-linked mCherry without sgRNA expression cassette.	7,872	This study
	sgRNA2/DCD2	sgRNA2 expression cassette and double cut donor 2 with undivided backbone.	4,909	This study
AIO system	AIO2	Cas9 2A peptide-linked mCherry, sgRNA2 expression cassette, and double cut donor 2 with undivided backbone.	10,773	This study
MC system (Divided backbone)	sgRNA1/DCD1	sgRNA1 expression cassette and double cut donor 1 with divided backbone.	5,144	This study
	sgRNA2/DCD2	sgRNA2 expression cassette and double cut 2 with divided backbone.	4,916	This study
PTG strategy	PTG/Cas9-2A-mCherry	Cas9 2A peptide-linked mCherry without specific targeting sgRNA expression via PTG strategy.	9,536	This study
	PTG/DCD1	Double cut donor 1 and sgRNA	5,391	This study

	expression without specific targeting via PTG strategy with divided backbone.		
PTG/DCD2	Double cut donor 2 and sgRNA expression without specific targeting via PTG strategy with divided backbone.	5,163	This study
PTG1_sgRNA1/Cas9-2A-mCherry	sgRNA1 expression cassette with PTG strategy (tRNA-sgRNA1-tRNA) and Cas9 2A peptide-linked mCherry.	9,538	This study
PTG2_ sgRNA1/Cas9-2A-mCherry	sgRNA1 expression cassette with PTG strategy (tRNA-sgRNA1-tRNA-sgRNA1-tRNA) and Cas9 2A peptide-linked mCherry.	9,711	This study
PTG1_ sgRNA2/Cas9-2A-mCherry	sgRNA2 expression cassette with PTG strategy (tRNA-sgRNA2-tRNA) and Cas9 2A peptide-linked mCherry.	9,538	This study
PTG2_ sgRNA2/Cas9-2A-mCherry	sgRNA2 expression cassette with PTG strategy (tRNA-sgRNA2-tRNA-sgRNA2-tRNA) and Cas9 2A peptide-linked mCherry.	9,711	This study
PTG1_ sgRNA1/DCD1	sgRNA1 expression cassette with PTG strategy (tRNA-sgRNA1-tRNA) and double cut donor 1 with divided backbone.	5,396	This study
PTG2_sgRNA1/DCD1	sgRNA1 expression cassette with PTG strategy (tRNA-sgRNA1-tRNA-sgRNA1-tRNA) and double cut donor 1 with divided backbone.	5,569	This study
PTG1_sgRNA2/DCD2	sgRNA2 expression cassette with PTG strategy (tRNA-sgRNA2-tRNA) and double cut donor 2 with divided backbone.	5,168	This study
PTG2_sgRNA2/DCD2	sgRNA2 expression cassette with PTG	5,341	This study

strategy (tRNA-sgRNA2-tRNA-sgRNA2-tRNA) and double cut donor 2 with divided backbone.

Supplementary Table S4. DNA brick information for USER cloning

Plasmid name	DNA Brick information	PCR template
DCD1	Homology left	Genomic DNA of monitoring CHO-K1
	Human EF-1α promoter	pBudCE4.1 [Life Technologies]
	Homology right	Genomic DNA of monitoring CHO-K1
	Backbone (AmpR, Ori)	pcDNA 3.1(+) [Life Technologies]
DCD2	Homology left	Genomic DNA of monitoring CHO-K1
	Human EF-1α promoter	pBudCE4.1 [Life Technologies]
	Homology right	Genomic DNA of monitoring CHO-K1
	Backbone (AmpR, Ori)	pcDNA 3.1(+) [Life Technologies]
sgRNA2/DCD2 (Original backbone)	U6-sgRNA2-terminator	sgRNA2/Cas9-2A-mCherry (this study)
	Double cut donor 2	DCD2 (this study)
	Backbone (AmpR, Ori)	pcDNA 3.1(+) [Life Technologies]
MC_Cas9-2A-mCherry	CMV promoter	pEGFP-C1 [Clontech]
	Cas9-2A-mCherry	Cas9-2A-mCherry (Addgene #64324)
	BGH pA	Cas9-2A-mCherry (Addgene #64324)
	Backbone (AmpR, Ori)	pcDNA 3.1(+) [Life Technologies]
AIO2	Double cut donor 2- U6-sgRNA2-terminator	sgRNA2/DCD2 (this study)
	CMV promoter	pEGFP-C1 [Clontech]
	Cas9-2A-mCherry-BGH pA	Cas9-2A-mCherry (Addgene #64324)
	Backbone (AmpR, Ori)	pcDNA 3.1(+) [Life Technologies]
sgRNA1/DCD1	U6-sgRNA1-terminator	sgRNA1/Cas9-2A-mCherry (this study)
	Backbone (AmpR)	pcDNA 3.1(+) [Life Technologies]
	Double cut donor 1	DCD1 (this study)

	Backbone (Ori)	pcDNA 3.1(+) [Life Technologies]
sgRNA2/DCD2 (Divided backbone)	U6-sgRNA2-terminator	sgRNA2/Cas9-2A-mCherry (this study)
	Backbone (AmpR)	pcDNA 3.1(+) [Life Technologies]
	Double cut donor 2	DCD2 (this study)
	Backbone (Ori)	pcDNA 3.1(+) [Life Technologies]
PTG/DCD1	U6-tRNA-Bbs1 recognition site-gRNA scaffold-tRNA-terminator	PTG/Cas9-2A-mCherry (this study)
	Backbone (AmpR)	pcDNA 3.1(+) [Life Technologies]
	Double cut donor 1	DCD1 (this study)
	Backbone (Ori)	pcDNA 3.1(+) [Life Technologies]
PTG/DCD2	U6-tRNA-Bbs1 recognition site-gRNA scaffold-tRNA-terminator	PTG/Cas9-2A-mCherry (this study)
	Backbone (AmpR)	pcDNA 3.1(+) [Life Technologies]
	Double cut donor 2	DCD2 (this study)
	Backbone (Ori)	pcDNA 3.1(+) [Life Technologies]

REFERENCES

[1] Feichtinger, J.; Hernández, I.; Fischer, C.; Hanscho, M.; Auer, N.; Hackl, M.; Jadhav, V.; Baumann, M.; Krempl, P. M.; Schmidl, C.; Farlik, M.; Schuster, M.; Merkel, A.; Sommer, A.; Heath, S.; Rico, D.; Bock, C.; Thallinger, G. G.; Borth, N. Comprehensive genome and epigenome characterization of CHO cells in response to evolutionary pressures and over time. *Biotechnol. Bioeng.* **2016,** 113, 2241–2253.

[2] Hernandez, I.; Dhiman, H.; Klanert, G.; Jadhav, V.; Auer, N.; Hanscho, M.; Baumann, M.; Esteve-Codina, A.; Dabad, M.; Gómez, J.; Alioto, T.; Merkel, A.; Raineri, E.; Heath, S.; Rico, D.; Borth, N. Epigenetic regulation of gene expression in Chinese Hamster Ovary cells in response to the changing environment of a batch culture. *Biotechnol. Bioeng.* **2019**, 116, 677–692.

[3] Chu, V.; Weber, T.; Wefers, B.; Wurst W.; Sander S.; Rajewsky K.; Kühn R. Increasing the efficiency of homology-directed repair for CRISPR-Cas9-induced precise gene editing in mammalian cells. *Nat Biotechnol* **2015,** 33, 543–548.

[4] Shin, S.W.; Lee, J.S. CHO Cell Line Development and Engineering via Site-specific Integration: Challenges and Opportunities. *Biotechnol. Bioproc. E.* **2020**, 25, 633–645.
